# Parents’ and teachers’ perspectives on the barriers to spectacle uptake among learners in the Free State, South Africa

**DOI:** 10.4102/hsag.v31i0.3192

**Published:** 2026-04-16

**Authors:** Xolani Nyathela, Urvashni Nirghin, Naimah Ebrahim Khan

**Affiliations:** 1 Discipline of Optometry, College of Health Sciences, University of KwaZulu-Natal, Durban, South Africa

**Keywords:** barriers, uptake, eye care, services prevalence, uncorrected refractive error

## Abstract

**Background:**

Parents’ and teachers’ awareness of their children’s visual challenges is critical in curbing the deleterious effects of uncorrected refractive errors (UREs), especially on their learning capabilities.

**Aim:**

This study aimed to determine the barriers to the uptake of spectacles according to the parents or guardians and teachers of high school learners in the Free State province.

**Setting:**

Free State province, South Africa.

**Methods:**

A quantitative, descriptive, cross-sectional study using a survey method was conducted among teachers and parents of learners diagnosed with UREs during earlier phases of the research, which are not reported in this publication. Participants self-administered questionnaires adapted to the Free State province.

**Results:**

A total of 132 parents and 23 teachers participated in this study. Red eyes were identified as an eye-related problem by 20.5% parents and 39.1% teachers, double vision by 24.2% parents and 30.4% teachers, while tearing eyes was identified by 30.4% parents and 39.1% teachers. About 60% parents did not know how far optometrists were, 55.7% were unemployed, and 3.8% had healthcare insurance. Only 8.8% and 4.4% of teachers associated spectacle wear non-compliance with lower self-confidence and poorer academic performance, respectively. A total of 9.1% parents and 8.7% teachers perceived spectacles as bad for the eyes. Also, 68.2% parents had never taken their children for an eye examination before.

**Conclusion:**

Parents’ and teachers’ lack of awareness and/or knowledge of eye-related problems adversely impacted the uptake of spectacles.

**Contribution:**

The affordability, availability, and accessibility of eye care services presented as obstacles to the uptake of spectacles for parents.

## Introduction

Caregivers’ and teachers’ inability to identify children’s visual challenges, compounded by children’s failure to report such challenges, facilitated the perpetuation of uncorrected refractive error (URE) (Alrasheed 2021). This failure was attributed to poor eye care literacy by school community members (Alrasheed 2021; Alrasheed, Mohamed & Alluwimi 2024; Alsaqr 2023; Du et al. 2022). Du et al. (2022) found that the uptake of eye care services depended on caregivers being informed by either their children or teachers; however, neither teachers nor children were wiser about eye-related problems. Therefore, when poor vision is neither reported nor noticed, URE treatment may be delayed, causing heightened cognitive and emotional stress, academic and non-academic frustration, such as playing in groups (Chan, Singer & Naidoo 2020; Magakwe, Hansraj & Xulu-Kasaba 2022; Ntodie et al. 2025; Zhou et al. 2024). Consequently, URE is associated with increased chances of dropping out of school, resulting in limited education, which adversely impacts employment opportunities, socioeconomic status, psychological well-being, and the quality of life (Asare & Morjaria 2021; Cao et al. 2022; Chan et al. 2020; Govender-Poonsamy et al. 2023; Huda, Ramzan & Imran 2021; Latif et al. 2022; Magakwe et al. 2022; Yang et al. 2021; Zhou et al. 2024). These consequences are plausible because vision is used up to 80% of the time during learning activities (Chan et al. 2020; Onyeahiri, Omere & Kyamru 2024). The URE has the greatest burden of disease among ocular conditions; hence, it is classified as a public health concern (Govender-Poonsamy et al. 2023; Magakwe et al. 2022; Magakwe, Xulu-Kasaba & Hansraj 2020; Yang et al. 2021; Zhou et al. 2024).

Teachers’ knowledge and/or awareness of common eye problems is indispensable in the eradication of preventable and avoidable blindness (Alemayehu, Belete & Adimassu 2018). Teachers’ well-established role as health educators, based on their contact time with children and positive attitudes, especially in under-resourced communities, can initiate a referral for eye examination (Alemayehu et al. 2018). The uptake of eye care services is well-documented to be imperative for the elimination of avoidable blindness among the litany of problems associated with compromised vision (Alrasheed, Naidoo & Clarke-Farr 2016; Alrasheed et al. 2018b; Li et al. 2010). However, caregivers’ perceptions, beliefs, and attitudes towards spectacles, which are influenced by their bias, are critical in the acquisition and use of spectacles (Alrasheed 2021; Alrasheed et al. 2016, 2024; Alrasheed, Naidoo & Clarke-Farr 2018a; Li et al. 2010).

Enabling factors, not exhaustive of healthcare insurance, income, wealth, and urban residence, were associated with eye care services uptake in South Africa (Akuffo et al. 2020). Children who were not covered by healthcare insurance were five times more likely to be at risk of unmet eye care needs (Alrasheed et al. 2016). Conversely, only 15.7% of the South African population had healthcare insurance in 2024, which enabled the accessibility of private health and eye care healthcare (Wolvaardt, Stein & Mumbauer 2025). According to Malakoane et al. (2020), healthcare in South Africa is divided into private and public sectors, with the latter funded by the government. Moreover, about 80% of the Free State province population is dependent on the failing public health sector (Malakoane et al. 2020). This dependence on the unavailable public healthcare services was attributed to the remnants of apartheid (Malakoane et al. 2020). Apartheid ensured that the country’s majority and native groups were socially, politically, and educationally segregated, and economically excluded; hence, the high unemployment rate (Malakoane et al. 2020; Statistics South Africa 2021, 2025).

Even though South Africa is ranked as an upper-middle-income country (World Bank 2025), it has arguably the worst inequality in the world and high unemployment rates (Makgetla 2020; Statistics South Africa 2021, 2025).

**FIGURE 1:**
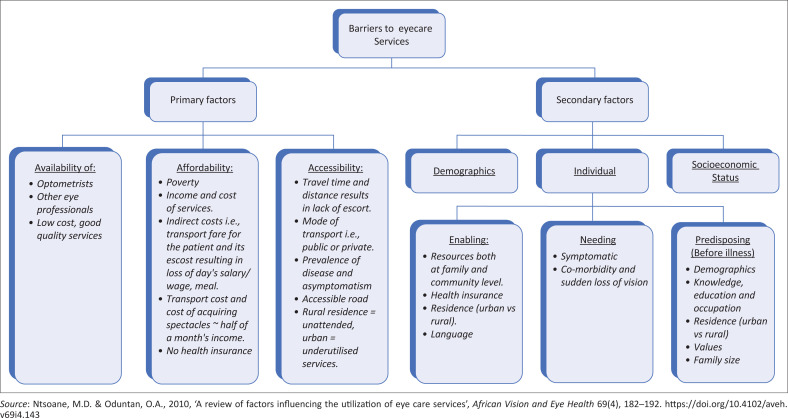
Barriers to the uptake of eye care services.

The cost of spectacles in developing countries was reported by several studies as the main barrier to the uptake of spectacles (Alrasheed 2021; Alrasheed et al. 2018a, 2024; Dhirar et al. 2020; Ezinne et al. 2020). It was established that the cost of the actual eye care services and cost-related factors make spectacles unaffordable, thus inaccessible (Alrasheed 2021; Alrasheed et al. 2018a, 2024; Magakwe et al. 2022). This is despite URE being the simplest and most affordable anomaly to diagnose and manage (Govender-Poonsamy et al. 2023; Magakwe et al. 2020). Figure 1 illustrates the barriers to the uptake of eye care services; however, the literature is limited regarding barriers from teachers’ perspectives.

In South Africa, there was an increase in the number of registered optometrists, from 3697 in 2016 to 4040 in 2024 (Health Professions Council of South Africa 2024; Maake & Moodley 2018). However, the number of these practitioners working in the public sector, which serves an estimated 80% of South Africans, has remained obstinately low (Maake & Moodley 2018; Makgetla 2020). Anecdotal evidence revealed that some government hospitals in the Free State operate with only ophthalmic nurses, some with no optometric services, while others do not dispense spectacles. This anecdotal evidence was corroborated by Malakoane et al. (2020) who discovered the exodus of healthcare professionals, inadequate financing, and dilapidated infrastructure. In addition, the public healthcare sector in the Free State province had poor healthcare quality because of fragmented service delivery and poor leadership (Malakoane et al. 2020). This resulted in a lack of trust and the non-utility of public health facilities by communities (Malakoane et al. 2020). To supplement the shortage of healthcare services and alleviate the burden of illness among learners by offering, among others, vision screening, the Integrated School Health Programme (ISHP) was launched (Department of Health & Department of Basic Education 2012). Literature is void of barriers to the uptake of spectacles among learners from the perspective of parents/ guardians and teachers in the Free State, hence this study.

## Research methods and design

### Study design

A quantitative, descriptive, cross-sectional study using a survey method was conducted among teachers and parents of learners diagnosed with UREs during earlier phases of the research, which are not reported in this publication. Cross-sectional surveys provide insights and comprehension of the problems encountered by populations of interest (Maier et al. 2023). Similarly, this study sought to understand barriers to the uptake of spectacles from the perspective of parents and teachers.

### Setting

The Free State province, a geographical area of 129 825 km^2^, is one of the nine South African provinces (Adeola et al. 2021). Quintiles one to five categories were developed by the democratic dispensation to redress apartheid-caused educational inequalities (Ogbonnaya & Awuah 2019). Because children from disadvantaged backgrounds are more likely to suffer from undiagnosed health problems (Dlamini & Mbonigaba 2024), this study focused on Q1–Q3 high schools in the Free State province. According to Ogbonnaya and Awuah (2019) Q1–Q3 schools cater for poor communities with Q1 classified as the poorest quintile.

### Study population

A total of 193 quintiles one to three (Q1–Q3) high schools in the Lejweleputswa, Motheo, Thabo Mofutsanyane, and Xhariep districts of the Free State province were listed (Free State Department of Education 2021). A total of 132 out of 233 parents/guardians and 23 out of 255 teachers participated in this study.

### Sampling

A multistage stratified random sampling method was used to select 868 learners from 51 high schools who participated in the prevalence of URE study (Nyathela, Nirghin & Ebrahim Khan 2025). The study unveiled a prevalence of URE of 27.1% (*n* = 233) (Nyathela et al. 2025). Subsequently, 233 parents/guardians of the learners identified with URE were asked to complete self-administered questionnaires. In addition, 255 class teachers (one per grade) from the same 51 schools were invited to complete the self-administered questionnaires. A feasibility study was undertaken in a conveniently selected Q2 school; however, the results of the study were excluded from the main results.

### Data collection and instrument

The parents’/guardians’ questionnaire was adapted from previous studies (Alrasheed et al. 2018a; Li et al. 2010), while the teacher’s questionnaire was adapted from Alemayehu et al. (2018) and Li et al. (2010). A language practitioner translated the English version of the questionnaire into Sesotho and Afrikaans, the other commonly spoken languages in the province.

Self-administered questionnaires were employed to determine the local barriers. They are frequently used to solicit information about participants’ knowledge, attitudes and perceptions, and in this study, towards eyecare services (Saunders & Kulchitsky 2021). These surveys are easy to administer, cost-effective, and more reliable compared to interviews (Saunders & Kulchitsky 2021).

All the questions included in the questionnaires were closed-ended except the last question. An open-ended question was included to help the researcher understand what participants would like to know concerning their children’s eyes. The teacher questionnaire had 17 questions and parents’/guardians’ questionnaire had 24 questions, requiring 5 min – 7 min to complete the questionnaire. The questionnaires were divided into five and four sections for parents/ guardians and teachers, respectively. Unlike the teacher’s questionnaire, the parents’ questionnaire covered affordability questions. Teachers’ questionnaire included years of teaching experience and awareness of the ISHP. Both parents/guardians and teachers were asked to identify (select) eye-related signs and symptoms, remedies to these problems, where eye care services are accessed, and how far eye care facilities were from home or school.

### Data management and analysis

Data were captured on Statistical Package for Social Sciences version 27 (IBM Corporation, Armonk, NY, United States) in consultation with a statistician. Data were analysed using descriptive statistics, distribution tables and proportions. The distribution of presenting barriers was tabulated mostly by gender. Categorical variables were summarised using frequency distribution tables and proportions.

### Ethical considerations

Ethical clearance to conduct this study was obtained from the Biomedical Research and Ethics Committee of the University of KwaZulu-Natal. The ethical clearance number is BREC/00005522/2023. The Free State Department of Education and individual school principals were approached for the granting of access and permission to utilise school premises. This study adhered to the principles of Good Clinical Practice (GCP) and the Declaration of Helsinki.

Permission to access the schools was granted by the Free State Department of Education on 28 March 2023, subject to the condition that access would be granted only after school hours. One of the researchers travelled to each of the schools from 27 July 2023 to 20 September 2023. Class teachers were given the questionnaires to complete, and learners were sent home with the questionnaires for the parents/guardians to complete and return to the liaison teacher the following day. Each participant was given a study information document, informing them of the purpose of the study and what the study entails. Signed written consent forms were a prerequisite for participation. Voluntary participation and withdrawal from further participation at any given time without any punitive measure were mentioned to participants as their rights. Confidentiality of data was maintained, and the identification of the participants was not presented in the results.

## Results

### Demographics

A total of 132 parents/guardians and 23 teachers returned their questionnaires. This translated to a response rate of 56.7% and 9% for parents/guardians and teachers, respectively. Most of the parents were black people 96.2% (*n* = 127) and female 71.2% (*n* = 94), while 3.8% (*n* = 5) were mixed-race people. Most caregivers (78%, *n* = 103) had secondary education compared to the 2.3% (*n* = 3) with no formal education. Similarly, 91.3% (*n* = 21) of the teachers were black people, and 65.2% (*n* = 15) were female, with 4.4% (*n* = 1) of each being Caucasian and mixed-race teachers.

A total of 82.4% (*n* = 114) of caregivers were aware that their children had eye problem(s) compared to the 12.9% (*n* = 17) who were unaware. Most caregivers noticed squinting (72.1%, *n* = 93), followed by itchy (49.6%, *n* = 64) and red eyes (39.5%, *n* = 51). Subsequently, 92.4% (*n* = 122) indicated that their children had complained of eye-related problems, while 6.1% (*n* = 8) did not know. Participants were asked to identify eye-related signs and symptoms (Table 1).

**TABLE 1:** Responses to the identification of signs and symptoms according to parents (*N* = 132) and teachers (*N* = 23).

Signs and symptoms	Parents		Teachers	
	Frequency (*n*)	%	Frequency (*n*)	%
Blurry vision	102	77.3	14	60.9
Double vision	32	24.2	7	30.4
Eye strain	67	50.8	8	34.8
Headaches	65	49.2	12	52.2
Itchy eyes	53	40.2	14	60.9
Red eyes	27	20.5	9	39.1
Teary eyes	46	30.4	9	39.1
Other	7	5.3	2	8.7
**Child classroom behaviour**				
Struggle to see the blackboard	N/A	N/A	14	60.9
Poor academic performance	N/A	N/A	14	60.9
Can’t write in straight lines	N/A	N/A	10	43.5
Limited attention span	N/A	N/A	9	39.1
Low self-esteem/ confidence	N/A	N/A	8	34.8
Withdrawn behaviour	N/A	N/A	4	7.4

N/A, Not applicable.

**FIGURE 2:**
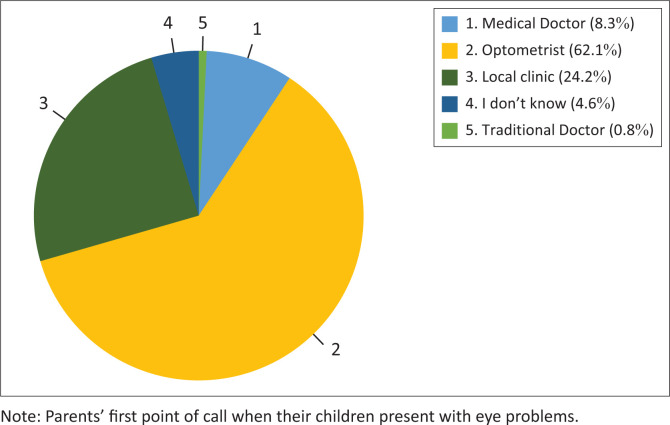
Parents’/guardians’ (*N* = 132) responses to the practitioner they would attend to when their children experience eye problems.

Figure 2 provides parents’ responses regarding the practitioner they take their children to when they have eye problems, indicating the availability of eye care services.

Most teachers (56.5%, *n* = 13) indicated that they advised the caregivers to take the child to healthcare facilities or move the child to the front of the classroom (17.4%, *n* = 4) when they notice eye-related problems. Furthermore, 13% (*n* = 3) either do nothing or instruct the child to stop rubbing their eyes. More than half of teachers (52.2%, *n* = 12) did not know about ISHP, with 17.4% (*n* = 4) indicating that ISHP was operational.

A total of 69.6% (*n* = 16) of teachers had previously examined their eyes. The results of the participants’ responses are summarised in Table 2. The acceptability questions included the perceived effects of spectacles and caregivers’ approval of spectacles.

**TABLE 2:** The acceptability responses of the parent/guardian (*N* = 132) and teacher (*N* = 23) participants.

Question	Parents		Teachers	
	Frequency (*n*)	%	Frequency (*n*)	%
**What are the effects of spectacle wear non-compliance?**				
No effects (impact)	32	24.2	1	4.4
It would worsen the child’s vision	55	41.7	19	82.6
Poor academic performance	24	18.2	1	4.4
Daily tasks will be impacted	10	7.6	-	-
It would affect their self-confidence	-	-	2	8.8
**Are spectacles only for adults?**				
Yes	4	3.0	1	4.4
No	125	94.7	22	95.7
I do not know	3	2.3	-	-
**Are spectacles bad for the eyes (can they weaken the eyes)**				
Yes	12	9.1	2	8.7
No	93	70.5	20	87
I do not know	27	20.5	1	4.4
**If your child were to be prescribed spectacles, would you allow him or her to wear them?**				
Yes	130	98.5	N/A	N/A
No	-	-	N/A	N/A
I do not know	2	1.5	N/A	N/A
**Please provide a reason for the question above**				
Spectacles will improve the child’s vision and concentration	128	97	N/A	N/A
My child will be reliant on spectacles	3	2.3	N/A	N/A
Spectacles will make my child look unattractive	1	0.8	N/A	N/A

N/A, Not applicable.

A total of 60.6% (*n* = 80) of caregivers did not know how far away optometric services were from their residences, while 19.7% (*n* = 26) indicated more than 20 km away. As a result, 5.3% (*n* = 7) took their children for eye examination in the last 2 years (Table 3). More than 83% of parents had either never taken their children for an eye examination before (68.2%, *n* = 90) or had done so more than 5 years ago (15.2%, *n* = 20) (Table 3).

**TABLE 3:** A summary of the accessibility of eye care services according to parents/guardians (*N* = 132) and teachers (*N* = 23).

Question	Parents†		Teachers‡	
	Frequency (*n*)	%	Frequency (*n*)	%
Close (< 10 km)	13	9.9	7	30.4
Far (11 km – 20 km)	13	9.9	6	26.1
Very far (> 20 km)	26	19.7	4	17.4
I do not know	80	60.6	6	26.1
**When last did you take your child for an eye test?**				
Never	90	68.2	N/A	N/A
More than 5 years	20	15.2	N/A	N/A
3–5 years	15	11.4	N/A	N/A
0–2 years	7	5.3	N/A	N/A

† , How far away is an optometrist from where you stay?;

‡ , How far away is an optometrist from the school?

N/A, Not applicable.

A total of 58.3% (*n* = 77) indicated that their inability to take children for an eye examination, while 14.4% (*n* = 19) were uncertain. Most of the caregivers (55.7%, *n* = 73) were unemployed, 31.8% (*n* = 42) were on state social grants, and 12.1% (*n* = 16) were employed. Moreover, 3.8% (*n* = 5) of the caregivers had health insurance in contrast to the 96.2% (*n* = 127) who did not. Three-quarters perceived spectacles as expensive, 3.8% (*n* = 5) did not hold this view, and 19.7% (*n* = 26) did not know.

## Discussion

The aim of this study was to determine the barriers to the uptake of spectacles from the perspective of caregivers and teachers in the Free State province. In summary, affordability, which is the ability of people to incur the cost of the actual healthcare services and the cost of other services incurred while seeking healthcare, influences the accessibility of eyecare services (Alrasheed et al. 2016, 2018a; Dhirar et al. 2020; Ezinne et al. 2020). Furthermore, accessibility of eyecare services is noted as the travel time to reach the nearest eyecare facility, closely linked to the residence, and the availability of transportation, including roads to access facilities (Alrasheed 2021). The existence of well-maintained infrastructure and practitioners who offer eyecare services is termed availability (Alrasheed 2021). Not exhaustive of compliance with spectacle wear, cultural beliefs, perceptions, practitioner behaviour and mistrust, gender, stigmas, and misconceptions formed part of the acceptability (Alrasheed et al. 2016, 2018a; Ezinne et al. 2020). Eye care literacy, that is, knowledge and awareness of eye-related signs and symptoms, what eye care services are available, and where they are available, which would enable communities to seek eye care expeditiously (Ezinne et al. 2020).

Participants in this study are unaware of eye problems. Only 61% of teachers identified blurry vision, 22% indicated squinting as an eye-related problem, while 40% failed to associate the struggle to see the blackboard with poor academic performance. Behavioural anomalies such as poor academic performance, limited attention span, low self-confidence/ esteem, and not writing in straight lines are exhibited by learners with URE (Chan et al. 2020). However, only 4.4% and 8.7% of teachers correlated spectacle non-compliance with substandard academic performance and low self-esteem/confidence, respectively. This highlighted that teachers in this study do not fulfil their role as health educators (Alemayehu et al. 2018). Not absolving parents/guardians from their responsibility towards their children, but teachers are better positioned to notice eye-related signs, symptoms, and behaviour (Chan et al. 2020; Govender-Poonsamy et al. 2025). This is because of the contact time they have with learners engaging in visually demanding tasks (Chan et al. 2020; Govender-Poonsamy et al. 2025).

Notwithstanding the above, a quarter of caregivers did not correlate the use of spectacles with alleviating visual challenges, and 13% were unaware of what could relieve eye problems. Some parents indicated good sleep, nutritious meals, and spontaneous resolution as remedies to eye problems, similar to other studies (Alrasheed et al. 2016; Li et al. 2014). The lack of awareness of eye-related signs and symptoms was one of the leading barriers in several studies (Alrasheed 2021; Alrasheed et al. 2016, 2018a, 2024). Poor health literacy is attributed to several factors, including the lack of eye care services and interventions such as health education programmes (Paudel et al. 2019, 2022). In the current study, these factors, together with rural residency and affordability, contributed to the poor health literacy.

In this study, about 10% of caregivers perceived spectacles as bad for the eyes, citing that spectacles would deteriorate eyes; however, this was less than the 78.6% in Sudan (Alrasheed et al. 2018a).

According to Alrasheed et al. (2016), 21.4% of parents sought eye care from traditional doctors compared to 1% in this study, possibly due to cultural differences in the studies. Caregivers indicated that they would allow their children to wear their spectacles, demonstrating high acceptability of spectacles. Conversely, caregivers in other studies dissuaded their children from wearing their spectacles, especially in public, as spectacles were perceived as signs of disability (Alrasheed et al. 2018a; Ezinne et al. 2020).

The mistrust of healthcare workers by the public did not emerge as a barrier to the uptake of eyecare services in this study unlike in South Sudan (Alrasheed et al. 2018a). Furthermore, teacher participants demonstrated an interest in being trained to conduct vision screening similar to Ethiopian teachers (Alemayehu et al. 2018). Elsewhere in South Africa, teachers demonstrated good skills in conducting vision screening when trained (Govender-Poonsamy et al. 2025).

Dlamini and Mbonigaba (2024) found that low maternal education and low family socioeconomic status result in unmet health needs of a child. In this study, 96.2% caregivers, the majority of whom were female and without tertiary education, did not have healthcare insurance, in contrast to the 39% in Sudan (Alrasheed et al. 2016). A total of 72.7% parents did not have the financial ability to take their children for an eye examination. This study highlighted that learners in the Free State province are unlikely to have their eye care needs met because of affordability. Similarly, the cost involved in the utility of eye care services and the acquisition of spectacles was reported as the main barrier by several studies (Alrasheed et al. 2016, 2018a; Dhirar et al. 2020; Ezinne et al. 2020).

About 70% of parents never took their children for an eye examination, despite 82.4% of parents being aware of their children’s visual challenges. This could be attributed to the inaccessibility of eye care services, as only 10% and 30% of parents and teachers indicated proximity to eye care services. Also, almost 40% of parents would not take their children to an optometrist, i.e. a primary eye care provider (Willie 2023), possibly because of the unavailability of optometrists in their communities or local clinics. Together with the unaffordability of eye care services because of unemployment and no healthcare insurance, among others, eye care services were inaccessible and unavailable in the Free State province.

### Limitations

Since self-administered questionnaires were used, the study was subjected to self-report bias (Belihu et al. 2024).

This study used a combination of ‘yes-and-no’ and ‘choose all the applicable’ question types. It was implausible to solicit all pertinent answers with only the ‘yes-and-no’. This study was exclusive to the Free State province and high schools therefore the findings cannot be generalised to other provinces and all primary schools. There was a low response rate, limiting the overall generalisation of the results.

### Recommendations

Teachers’ poor awareness of eye care-related knowledge highlighted the need to include basic eye care knowledge in the curriculum offered to Bachelor of Education undergraduate students. Moreover, feasibility studies need to be undertaken to assess teachers’ competence in conducting vision screening in the Free State province. Also, there is a need for school-based eye care health promotion activities to ameliorate school communities’ knowledge of eye care. There is a need to undertake eye care health promotion campaigns at clinics, churches, public transport stations, and through mass media. The implementation of the ISHP policy needs to be re-strategised by the Departments of Health and Education for improved implementation.

## Conclusion

The poor eye care knowledge, together with inaccessible, unavailable and unaffordable eye care services, were displayed as barriers to the uptake of spectacles among learners in the Free State by the participants. This study highlighted that learners from no fee-paying schools in the Free State are prone to suffer the burden of URE, making them more susceptible to visual impairment and other eye care problems. Both parent and teacher participants presented a high acceptability of eye care services; however, poor eye care knowledge prevented what could otherwise be a much higher acceptability rate.
